# Clinical Comparison of Two Versions of a Commercial Artificial Intelligence System for Identifying Candidate Locations of Cerebral Aneurysms: Reduction and Characterization of False-Positive Findings

**DOI:** 10.3390/jcm15187259

**Published:** 2026-09-18

**Authors:** Masashi Kuwabara, Fusao Ikawa, Nami Saito, Antoine Choppin, Miwa Nishida, Daizo Ishii, Takeshi Hara, Shingo Matsuda, Nobutaka Horie

**Affiliations:** 1 Department of Neurosurgery, Graduate School of Biomedical and Health Sciences, Hiroshima University, 1-2-3 Kasumi, Minami-ku, Hiroshima 734-8551, Japan; m214028@gmail.com (M.K.); daiishii@hiroshima-u.ac.jp (D.I.); thara@hiroshima-u.ac.jp (T.H.); shima2da@hiroshima-u.ac.jp (S.M.); nobstanford@gmail.com (N.H.); 2 Department of Neurosurgery, Shimane Prefectural Central Hospital, Izumo 693-8555, Japan; 3 LPIXEL Inc., 1-6-1 Otemachi, Chiyoda-ku, Tokyo 100-0004, Japan; nami.saito@lpixel.net (N.S.); choppin@lpixel.net (A.C.); miwa.nishida@lpixel.net (M.N.)

**Keywords:** artificial intelligence, Brain Dock, cerebral aneurysm, false positive, magnetic resonance imaging

## Abstract

**Background/Objectives:** Despite high sensitivity in deep learning models for identifying candidate locations of unruptured cerebral aneurysms (UCAs), excessive false-positive (FP) findings and insufficient characterization of their anatomical distribution remain barriers to clinical integration. We evaluated a multi-stage deep learning framework for reducing anatomy-specific FPs in TOF-MRA-based UCA screening. **Methods:** This retrospective multicenter study analyzed TOF-MRA images from 404 scans across seven institutions. A baseline model (Model-A) was compared with an updated model (Model-B) incorporating a secondary cluster-filtering algorithm. Reference standards were established by expert reviewers. Aneurysm-wise sensitivity was compared using McNemar’s test, FPs per case using the Wilcoxon signed-rank test, and FP distributions by vascular location. **Results:** Aneurysm-wise sensitivity was 94.3% (231/245) for Model-A and 93.9% (230/245) for Model-B, with 1.16 and 0.879 FPs per case, respectively, representing a significant 24% FP reduction (*p* < 0.001). Per-aneurysm sensitivity did not differ significantly (McNemar’s test, *p* = 1.00). Model-B suppressed mimics in the internal carotid artery (ICA) and middle cerebral artery (MCA). Aneurysms were most frequent in the ICA (29%) and MCA (22%). Setting Model-A FPs as 100%, ICA and MCA accounted for 47.1% and 11.3% in Model-A and decreased to 34.5% and 7.7% in Model-B. In an exploratory regional analysis with Holm adjustment, FP reductions remained significant in the ICA (adjusted *p* < 0.001) and MCA (adjusted *p* = 0.003). **Conclusions:** A secondary cluster-filtering algorithm reduced FP candidate findings by 24% with similar sensitivity point estimates (paired difference −0.4 percentage points, 95% CI −3.1 to +2.1). Region-specific error analysis revealed the anatomical dependency of FP reduction. The impact on reading workflow and patient outcomes requires prospective evaluation.

## 1. Introduction

In adults, the prevalence of unruptured cerebral aneurysms (UCAs) has been reported to range from 2% to 6%, and the annual rupture rate in Japan is known to be approximately 0.95% [1,2,3,4]. Notably, the risk of aneurysmal rupture among Japanese individuals is approximately 2.8 times higher than that among Western populations, and these epidemiological characteristics underscore the importance of early detection of UCAs through the Brain Dock (brain check-up) system [5,6].

In Japan, the Brain Dock is a nationwide screening program supported by local governments that uses MRI and magnetic resonance angiography to detect asymptomatic cerebral infarction, unruptured cerebral aneurysms, and brain tumors at an early stage [6,7,8]. By facilitating early intervention and promoting lifestyle modification, the program aims to reduce the future risk of stroke and dementia. The Brain Dock is therefore regarded as a unique preventive medicine system in Japan [6].

The increasing volume of Brain Dock examinations has placed a substantial workload on radiologists, creating demand for automated support tools. Advances in cloud-based technology have facilitated the clinical introduction of artificial intelligence (AI)-assisted image diagnostic systems, and AI-based software for identifying candidate locations of UCAs has demonstrated high sensitivity in multiple studies [9,10,11,12,13,14,15,16]. However, high sensitivity is often accompanied by an increased number of false positives (FPs), which may offset the clinical benefits by increasing interpretive burden, unnecessary follow-up examinations, patient anxiety, and healthcare costs [9,13]. Consequently, improving AI algorithms to balance sensitivity and specificity remains an important challenge for their practical clinical application [9,12,13].

FPs in AI systems for detecting candidate sites of cerebral aneurysms commonly arise from anatomical structures such as arterial bifurcations, vascular infundibula, venous structures, and imaging artifacts [13,17]. Many existing models are optimized primarily for sensitivity, reflecting the clinical concern of missing aneurysms and the methodological difficulty of defining true-negative vascular findings [18]. As a result, conventional performance metrics may overestimate real-world usability, particularly in low-prevalence screening populations [9,18]. From a clinical perspective, reducing FPs is therefore not merely a technical refinement but a prerequisite for the safe and effective implementation of AI in routine practice [19,20]. Although recent studies have emphasized the gap between experimental AI performance and real-world deployment, systematic investigations focusing on FP reduction in identifying candidate locations of intracranial aneurysms remain limited [18,21]. Prior research has largely concentrated on network architecture or dataset expansion, with relatively little attention to post-processing strategies, multi-stage classification, or the analysis of qualitative changes in inference behavior accompanying algorithm refinement [22,23].

Beyond aneurysm detection itself, computer-assisted approaches are increasingly used across the aneurysm care pathway, including hemodynamic simulation and virtual stenting for treatment planning of intracranial aneurysms [24]. In this evolving ecosystem, screening tools such as candidate-location identification AI occupy the earliest step of the pathway, where the balance between sensitivity and false-positive burden most directly influences the number of individuals referred to downstream evaluation. Refining this first step is therefore a prerequisite for the coherent operation of subsequent computer-assisted aneurysm management.

Accordingly, the aim of this study was to investigate strategies for reducing false positives in MRI-based AI systems for identifying candidate locations of UCAs. By analyzing FP occurrence and comparing inference patterns between different model versions, we sought to improve the clinical usability and reliability of AI-assisted identification of candidate locations for aneurysms and to provide a practical framework for evaluating AI model refinement in real-world screening settings.

## 2. Materials and Methods

### 2.1. Ethics Statement

This research was conducted in accordance with the principles of the Declaration of Helsinki and complies with ethical guidelines for medical research. This study was approved by the Institutional Review Board of Shimane Prefectural Central Hospital (approval number: R25-006). As all individual data were anonymized and retrospectively collected, the requirement for informed consent was waived. This study followed the TRIPOD + AI reporting guidelines [25].

### 2.2. Datasets

**Figure 1:**
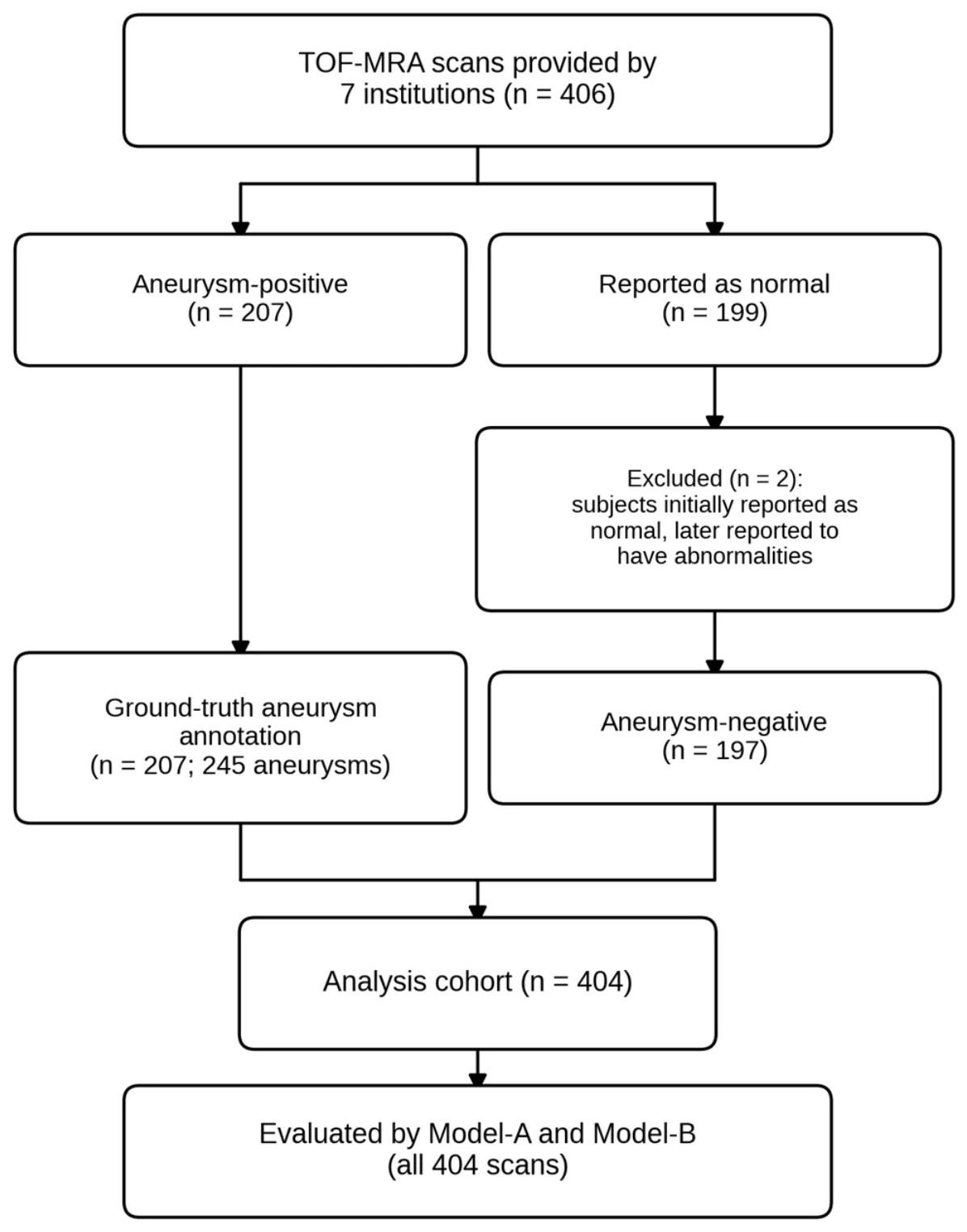
Study flow diagram. Of 406 TOF-MRA scans provided by the seven participating institutions, 2 were excluded because the originating institution subsequently reported abnormalities in subjects initially reported as normal, leaving an analysis cohort of 404 scans (207 aneurysm-positive, comprising 245 ground-truth aneurysms, and 197 aneurysm-negative), all of which were evaluated by both Model-A and Model-B.

We used time-of-flight (TOF) magnetic resonance angiography (MRA) images to evaluate the performance of algorithms for identifying candidate locations of aneurysms. A multicenter dataset was utilized for performance validation. The dataset comprised 404 MRA scans from 404 subjects, collected from seven institutions (hospitals or clinics). These 404 scans were derived from 406 scans initially provided by the participating institutions; 2 scans from subjects initially reported as normal were subsequently reported by the originating institution to have abnormalities and were therefore excluded (Figure 1). None of the cases in this dataset were used for model training, and all data were obtained from institutions different from those contributing to the training datasets of the evaluated models. The study size of 404 cases was determined based on the availability of eligible cases from participating centers during the study period and was considered adequate for the present evaluation of AI performance. The dataset was balanced to include approximately 50% cases with UCAs and 50% without. The approximately 1:1 ratio of aneurysm-positive to aneurysm-negative scans was chosen a priori to provide a sufficient number of aneurysms for aneurysm-wise sensitivity estimation and for stratified analyses by size, location, and field strength. This enriched design does not reflect the prevalence of UCAs in a screening population (approximately 2–6%), and prevalence-dependent quantities derived from this dataset should be interpreted accordingly. MRA scans were obtained using either 1.5 Tesla (T) or 3.0 T MRI scanners. Detailed information on the MRI scanners is provided in Supplementary Table S1.

The TOF-MRA scans analyzed in this study were acquired between November 2002 and August 2022. Eligible subjects were individuals who underwent brain screening (Brain Dock) at the participating institutions and whose TOF-MRA examinations were retrievable from the institutional archives. Aneurysm-positive and aneurysm-negative scans were included in approximately equal numbers by design; no further selection was applied within either group. No upper or lower age limit was applied. No explicit exclusion criterion was applied for previously treated aneurysms or prior intracranial surgery; such subjects were not within the scope of the data collection at the participating institutions. Apart from the 2 scans described above, no scan was excluded, and no exclusion was made on the basis of image quality (Figure 1). There were no missing imaging data or outcome annotations among the 404 included scans.

Ground-truth labels regarding the presence or absence of UCAs in each scan were provided by the original institutions. All aneurysms were inspected and annotated by expert reviewers, consisting of board-certified radiologists or neurosurgeons with at least 5 years of experience in diagnosing cerebral aneurysms. Each aneurysm was annotated in terms of its position, size, and anatomical location, and this information was used as the ground-truth for model evaluation (Table 1). Each scan was reviewed by one or two of three annotators, and an aneurysm was included in the ground truth if it was identified. The ground-truth annotations were established by the annotators who were blinded to the AI outputs. Disagreements were resolved by consensus discussion. Annotations were based on the TOF-MRA source images and maximum-intensity-projection reconstructions. The annotators knew only whether a scan had been reported as aneurysm-positive or aneurysm-negative, not the location or size of any aneurysm. All annotators were board-certified radiologists or neurosurgeons employed by medical or academic institutions. Aneurysms were identified and their three-dimensional extent assessed using both the axial images and maximum-intensity-projection reconstructions. To record the size, the annotating expert then drew an ellipse on a single representative axial slice to represent the extent of the aneurysm dome as assessed, and the length of the major axis of that ellipse was taken as the maximum diameter of the aneurysm.

In this study, only saccular aneurysms measuring 2 mm or larger were included as evaluation targets. Other vascular abnormalities such as dissecting aneurysms, fusiform aneurysms, and infundibular dilatations may have been present in the scans but were not included in the evaluation targets. Candidate locations produced at such lesions were therefore counted as false positives by definition.

### 2.3. Model Algorithms

Two AI-based deep learning models, referred to as Model-A and Model-B, were evaluated in this study. Both models were developed by LPIXEL Inc. (Tokyo, Japan) and released as part of their commercial software package (Medical Image Analysis Software EIRL aneurysm (Model-A, version 1.13.1; Model-B, version 2.0; approval number: 30100BZX00142000). The models are designed to detect candidate positions of UCAs from MRA scans in Digital Imaging and Communications in Medicine (DICOM) format.

The algorithms behind both models are described in the manufacturer’s documentation, which has been approved by the Pharmaceuticals and Medical Devices Agency (PMDA). Model-A, approved in 2021, is the initial version and serves as the baseline for comparison. Model-B, released in 2025, is an updated version developed to reduce the number of false positives (FPs) while maintaining sensitivity in identifying candidate locations of UCAs. Model-A utilizes a three-step processing pipeline to identify candidate locations of UCAs. First, vascular structures are extracted from the imaging data, and characteristic key points are identified. Next, for each key point, a small three-dimensional (3D) image patch is analyzed using two convolutional neural networks (CNNs), which compute a confidence score indicating the likelihood of an aneurysm. Finally, key points with high confidence scores are grouped into clusters, from which the final UCA candidate locations are determined. Model-B shares the first two steps with Model-A but differs in the final stage. Instead of using clustering alone, Model-B employs a separate model to evaluate clusters of key points and assign scores for the final UCA candidate selection. This refinement is intended to reduce FPs while preserving high accuracy in identifying candidate locations. Both models are designed to output up to four candidate UCA locations per MRA scan, limiting the number of candidate locations to four per case. Because the output is capped at four candidates per scan, per-scan false-positive counts are right-truncated by design.

### 2.4. Evaluation Methods

The annotated UCA positions were used as the ground truth for evaluating model performance. In cases where no aneurysm was present, the annotation “no aneurysm” served as ground truth. For each scan in the dataset, the models inferred candidate positions of UCAs.

A finding was considered a true positive (TP) when the Euclidean distance between the predicted point and the ground-truth aneurysm center was within a predefined tolerance distance. The tolerance distance was defined as the aneurysm radius derived from the ground-truth aneurysm size plus an additional 5 mm margin. This margin was introduced to account for multiple sources of spatial uncertainty inherent to point-based candidate location identification, including the spatial extent of the aneurysm, potential variability in manual annotation of the aneurysm center, and localization uncertainty of the identification model. Positional uncertainty of approximately 3 mm was allowed on each of these two sides. On the annotation side, the center of an aneurysm arising from a vessel wall cannot be indicated exactly, and the marked point may fall towards the lesion surface rather than its center. On the inference side, the models do not output lesion size, so an uncertainty of comparable magnitude was assumed on the basis of a typical aneurysm diameter. A 5 mm margin was adopted to accommodate these two sources of positional uncertainty. As a sensitivity analysis, all model outputs were re-scored with the margin set to 5, 4, and 3 mm, and sensitivity and FPs per case at each criterion are reported (Supplementary Table S6). In addition, the margin corresponds to the spatial scale used to visualize model findings in the clinical interface and approximates the practical tolerance applied when interpreting candidate aneurysm locations. Considering the spatial resolution of images and the inherent uncertainty in defining lesion centers in small vascular structures, this tolerance was adopted to provide a robust and clinically meaningful evaluation of performance. Similar spatial tolerance criteria have been adopted in prior studies evaluating point-based candidate location identification [9,13].

If an inferred position exceeded this threshold distance from all ground-truth aneurysms, it was counted as an FP. When multiple inferred positions were located within the threshold distance of a single aneurysm, only one was counted as a TP; the remaining positions within the threshold distance were counted as neither TP nor FP.

Model performance was evaluated aneurysm-wise, rather than per scan.

Sensitivity was calculated as:

Sensitivity = (Number of true positive findings)/(Total number of aneurysms)

False positives per scan (FPs/case) were calculated as:

FPs/case = (Total number of false positive findings)/(Total number of scans)

As a secondary measure of performance, the negative predictive value (NPV) was also calculated. In contrast to the aneurysm-wise sensitivity evaluation, NPV was evaluated per scan. A true negative (TN) scan was defined as a scan in which the model produced no UCA candidate positions and no aneurysms were annotated in the ground-truth. A false negative (FN) scan was defined as a scan in which the model produced no UCA candidates, even though at least one aneurysm was actually present.

Using these definitions, NPV was calculated as:

NPV = (Number of TN scans)/(Number of TN scans + Number of FN scans)

Because the negative predictive value depends on the prevalence of aneurysms in the population examined, the value reported here applies to the composition of this validation set (51.2% aneurysm-positive scans) and is not transferable to a screening population. It should be noted that false-negative scans refer to entire scans with no predictions, whereas for sensitivity, they refer to individual undetected aneurysms.

### 2.5. Inspection of False-Positive Findings

In this study, we used two models (Model-A and Model-B), with Model-B being a newly updated version. The purpose of the Model-B update was to reduce false-positive findings compared with Model-A. Therefore, in addition to a general FP evaluation of false-positive findings (FPs/case), we characterized the false-positive findings. FPs were classified according to the same vascular location categories used for ground-truth aneurysms (e.g., ICA, MCA). The anatomical location label of each false-positive finding was assigned by one of two board-certified neurosurgeons employed by medical or academic institutions, each with over 10 years of experience, who were not involved in the ground-truth annotation and were blinded to which model had generated each finding. To reduce the labeling workload, the location field was pre-filled using a separate model that infers the vascular territory from the coordinates of the finding; this model is distinct from the aneurysm-detection software evaluated in this study. The 404 scans were randomly divided into two non-overlapping subsets, one assigned to each reviewer. Findings from Model-A and Model-B were presented without any indication of their source. For both Model-A and Model-B, we compared the locations of the inspected false-positive findings with those of the confirmed true-positive inferences.

To further characterize FPs, the anatomical status of positions identified as FPs in a subset of 78 scans, randomly selected from the scans in which at least one false-positive finding was identified, was classified by the same two reviewers, each within their assigned scans, into four categories: normal anatomy, aneurysm or suspected aneurysm, other pathological conditions, and indeterminate. Because the evaluation targets were restricted to saccular aneurysms of 2 mm or larger, findings assigned to the category “aneurysm or suspected aneurysm” include lesions outside this definition and do not necessarily indicate an omission from the ground truth. In addition, as an exploratory analysis, the same reviewers classified each FP according to expert-perceived clinical acceptability, following a written annotation manual prepared before the review. An acceptable FP was defined as a model-generated finding considered clinically useful for diagnosis or clinical decision-making, that is, a finding the reviewer judged worth being alerted to in routine practice, such as a non-aneurysmal structure whose morphology warranted confirmation, whereas an unacceptable FP was defined as a model-generated finding that was not considered useful and was deemed unnecessary to report. Acceptability was judged individually for each finding and was not determined by the anatomical category assigned.

### 2.6. Statistical Analysis

All statistical analyses were performed using Python (version 3.10.13). Continuous variables are presented as mean ± standard deviation or median with interquartile range, as appropriate. Because paired comparisons were conducted between two models evaluated on the same patients and normality of paired differences was not assumed, nonparametric tests were used. A *p* value < 0.05 was considered statistically significant unless otherwise specified.

Sensitivity was calculated on a per-aneurysm basis for both Model-A and Model-B. Statistical significance was assessed using 95% confidence intervals (CI) calculated with the exact binomial method for sensitivity. The per-aneurysm sensitivities of Model-A and Model-B were compared using McNemar’s test for paired binary outcomes, with each aneurysm classified according to whether it was detected by each model. The paired difference in aneurysm-wise sensitivity between the models is reported with a score-based 95% confidence interval for paired proportions (Tango’s method). No non-inferiority margin was prespecified, and the comparison of sensitivities is descriptive.

The number of false positives (FPs) per case was compared between the two models using a two-sided Wilcoxon signed-rank test, with the alternative hypothesis defined as a difference in median false positives between the models. Because per-scan FP counts are integers, ties among the absolute differences are frequent and the exact null distribution implemented in SciPy, which assumes untied absolute differences, is not applicable. Exact two-sided *p* values were therefore computed from the conditional sign-flip distribution, enumerating all sign assignments given the observed absolute differences, with mid-ranks assigned to ties and zero differences excluded.

To assess regional dependency, the number of FPs was further analyzed for each vascular region as an exploratory analysis. For each region, paired comparisons between Model-A and Model-B were performed using a two-sided Wilcoxon signed-rank test; *p* values were adjusted for the eight regional comparisons using the Holm method. For the four principal aneurysm locations (ICA, MCA, ICPC, and ACOM), the observed FP distribution across locations was compared with the expected distribution derived from the ground-truth proportions using a global chi-square goodness-of-fit test for each model, followed, where significant, by post hoc two-sided exact binomial tests with Holm adjustment. Location-dependent sensitivity was tested with an exact test of the location × detection-status table (Fisher–Freeman–Halton test), with the exact *p* value obtained by complete enumeration of all tables with the observed margins.

The Wilcoxon signed-rank tests, chi-square goodness-of-fit tests, and exact binomial tests were implemented using the wilcoxon, chisquare, and binomtest functions from the scipy.stats module (SciPy, version 1.15.3); the exact test of the location × detection-status table was implemented by complete enumeration, as SciPy does not provide an exact test for r × c tables.

## 3. Results

We analyzed the dataset, as described in the Materials and Methods section. Of the 404 cases, 207 (51.2%) contained at least one UCA, accounting for 245 aneurysms in total, while the remaining 197 (48.8%) were aneurysm-free, consistent with the balanced study design. Among the 207 aneurysm-positive cases, 176 (85.0%) harbored a single aneurysm, 25 (12.1%) two, 5 (2.4%) three, and 1 (0.5%) four; multiple aneurysms were therefore present in 31 cases (15.0%), with a maximum of four in a single case. Aneurysm-related characteristics, including size and location, are presented in Table 1.

### 3.1. Evaluation of Candidate Locations of UCAs

Candidate UCA positions were inferred using Model-A and Model-B, and compared against ground-truth annotations to evaluate performance in identifying candidate locations. Results are summarized in Table 2. Model-A achieved a sensitivity of 94.3% (95% CI [90.6, 96.8]) and an FP burden of 1.16 FPs per case. Model-B had a sensitivity of 93.9% (95% CI [90.1, 96.5]) and 0.879 FPs per case, corresponding to a 24% decrease relative to Model-A. The per-aneurysm sensitivity did not differ significantly between Model-A and Model-B (McNemar’s test, *p* = 1.00). The paired difference in aneurysm-wise sensitivity (Model-B minus Model-A) was −0.4 percentage points (95% CI, −3.1 to +2.1), based on four aneurysms detected only by Model-A and three detected only by Model-B. Because no non-inferiority margin was prespecified, these results should not be interpreted as establishing non-inferiority. The confidence interval is compatible with a sensitivity of Model-B up to 3.1 percentage points lower, or up to 2.1 percentage points higher, than that of Model-A. On a per-scan basis, at least one aneurysm was detected in 199 of the 207 aneurysm-positive scans by each model, corresponding to a scan-wise sensitivity of 96.1% for both Model-A and Model-B, with no significant difference (McNemar’s test, *p* = 1.00). The reduction in FPs was statistically significant according to the Wilcoxon signed-rank test (*p* < 0.001). When stratified by the presence of aneurysms, the number of FPs per scan was 1.183 for Model-A and 0.959 for Model-B in aneurysm-negative scans, and 1.140 and 0.802, respectively, in aneurysm-positive scans. Re-scoring under stricter spatial criteria yielded consistent results (Supplementary Table S6): aneurysm-wise sensitivity was 93.5% and 91.8% for Model-A and 93.1% and 90.6% for Model-B under the radius + 4 mm and radius + 3 mm criteria, with FPs per case of 1.168 and 1.178 for Model-A and 0.884 and 0.899 for Model-B. At the scan level, the negative predictive value was 95.6% for Model-A and 94.1% for Model-B (Table 2). These values were obtained in a validation set comprising 51.2% aneurysm-positive and 48.8% aneurysm-negative scans.

### 3.2. Performance by Aneurysm Size

Performance in identifying candidate locations was further analyzed according to aneurysm size, with the results summarized in Table 1. Model-A correctly detected 231 out of 245 aneurysms, while Model-B detected 230. Sensitivity tended to be lower for small aneurysms, particularly those smaller than 3 mm in diameter. The smallest aneurysm correctly detected was 2 mm in diameter for both Model-A and Model-B, corresponding to the lower size limit of aneurysms included in this study. On the other hand, sensitivity was relatively high for medium and large aneurysms. However, it should be noted that the number of large aneurysms—defined as those measuring 10 mm or larger—was limited, with only seven cases in the dataset. As a result, statistical interpretation of performance in this size category remains limited.

### 3.3. Performance by Aneurysm Location

Performance was also analyzed according to aneurysm location, based on ground-truth annotations that specified the arterial segment of each aneurysm. The results by location are summarized in Table 1. In the anterior communicating artery (ACOM) region, there were 30 aneurysms in the dataset. Model-A correctly detected 28 of the 30 ACOM aneurysms, while Model-B detected 27.

The dataset contained 70 internal carotid artery (ICA), 44 internal carotid–posterior communicating artery (ICPC), and 55 middle cerebral artery (MCA) aneurysms; sensitivity at these locations is given in Table 1. Regions with fewer cases, such as the anterior cerebral artery (ACA), basilar artery (BA), and vertebral artery (VA), are also included in the analysis. However, due to the small sample sizes, particularly in these less common locations, caution is warranted when interpreting performance differences.

### 3.4. Performance by MRI Field Strength

Model performance was also examined with respect to the magnetic field strength of the MRI scanners, specifically comparing 1.5 T and 3.0 T systems. The numbers of scans by field strength were 260 at 1.5 T and 144 at 3.0 T. The numbers of aneurysms and correctly detected aneurysms categorized by field strength are presented in Table 1. In scans obtained with 1.5 T MRI systems, 177 and 176 aneurysms were detected by Model-A and Model-B, respectively. In 3.0 T scans, both models detected 54 aneurysms. Sensitivity was similar at 1.5 T and 3.0 T for both models. False-positive rates varied by field strength: for both models, the FP rate was higher at 3.0 T than at 1.5 T (Model-A, 1.46 vs. 1.00 FPs per scan; Model-B, 1.07 vs. 0.77). Within each field strength, Model-B significantly reduced false positives relative to Model-A (1.5 T: 1.00 to 0.77 FPs per scan, a 22.4% reduction; 3.0 T: 1.46 to 1.07, a 26.7% reduction; Wilcoxon signed-rank test, *p* < 0.001 for both), indicating a consistent false-positive reduction across field strengths (Supplementary Table S2).

To further examine whether detection sensitivity varied jointly with field strength and aneurysm size, per-aneurysm sensitivity was cross-tabulated by field strength and size category (Supplementary Table S3). The size-dependent pattern observed overall was preserved within each field strength: sensitivity was lowest for aneurysms smaller than 3 mm and reached or approached 100% for aneurysms of 3 mm or larger. At 1.5 T, sensitivity for aneurysms smaller than 3 mm was 85.7% (42 of 49) for Model-A and 83.7% (41 of 49) for Model-B, and was 93.2% or higher in every larger size category for both models. At 3.0 T, sensitivity for aneurysms smaller than 3 mm was 91.9% (34 of 37) for both models, and all aneurysms of 3 mm or larger were detected; however, the number of aneurysms in each size category at 3.0 T was small, with no aneurysm exceeding 10 mm and only four in the 5-to-10 mm category, so these estimates should be interpreted with caution. Within every combination of field strength and size category, the two models detected an essentially identical set of aneurysms, and at 3.0 T detection was identical across all size categories, consistent with the fully concordant detection at 3.0 T described above. Overall, the size-dependent pattern was present at both field strengths; sensitivity for aneurysms smaller than 3 mm was numerically lower at 1.5 T than at 3.0 T, although the small number of aneurysms imaged at 3.0 T limits interpretation of this difference. Per-institution performance is summarized in Supplementary Table S7.

### 3.5. Concordance of Detection Between the Two Models

To further characterize the difference in detection between the two models, we examined detection agreement at the level of individual aneurysms. The same 227 of the 245 aneurysms were correctly detected (true positives) by both models, and 11 were missed by both. The two models differed for only seven lesions: four aneurysms were true positives for Model-A but missed by Model-B, and three were true positives for Model-B but missed by Model-A, a net difference of one aneurysm.

The four aneurysms detected only by Model-A were a 2.3 mm ICA, a 2.6 mm ICPC, a 2.8 mm ACOM, and a 4.6 mm VA aneurysm. The three detected only by Model-B were a 2.3 mm MCA, a 2.5 mm VA, and a 5.6 mm MCA aneurysm. Most of these discordant lesions were smaller than 3 mm; the two exceptions were the 4.6 mm VA aneurysm detected only by Model-A and the 5.6 mm MCA aneurysm detected only by Model-B. All seven were imaged at 1.5 T, whereas detection at 3.0 T was fully concordant between the two models. The characteristics of these seven discordant aneurysms are summarized in Table 3.

### 3.6. Analysis of False-Positive Findings

**Figure 2:**
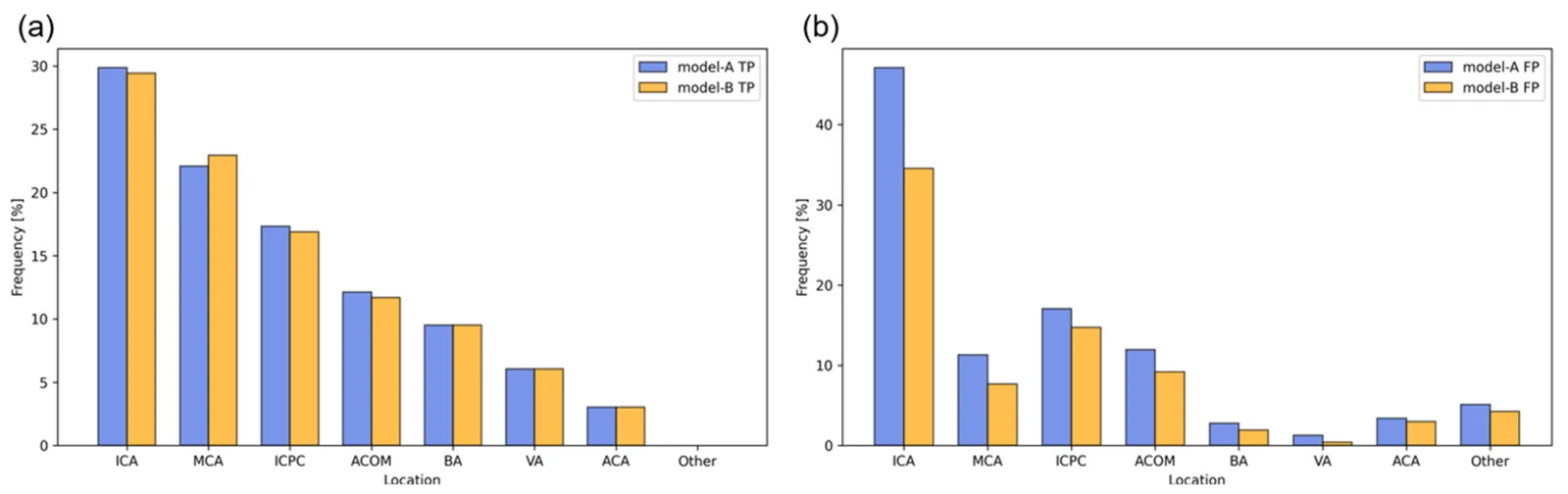
Distribution of inference locations. (**a**) Distribution of true-positive locations for Model-A and Model-B, expressed as percentages and normalized to the number of true positives detected by Model-A. (**b**) Distribution of false-positive locations for Model-A and Model-B, expressed as percentages and normalized to the number of false positives generated by Model-A.

**Figure 3:**
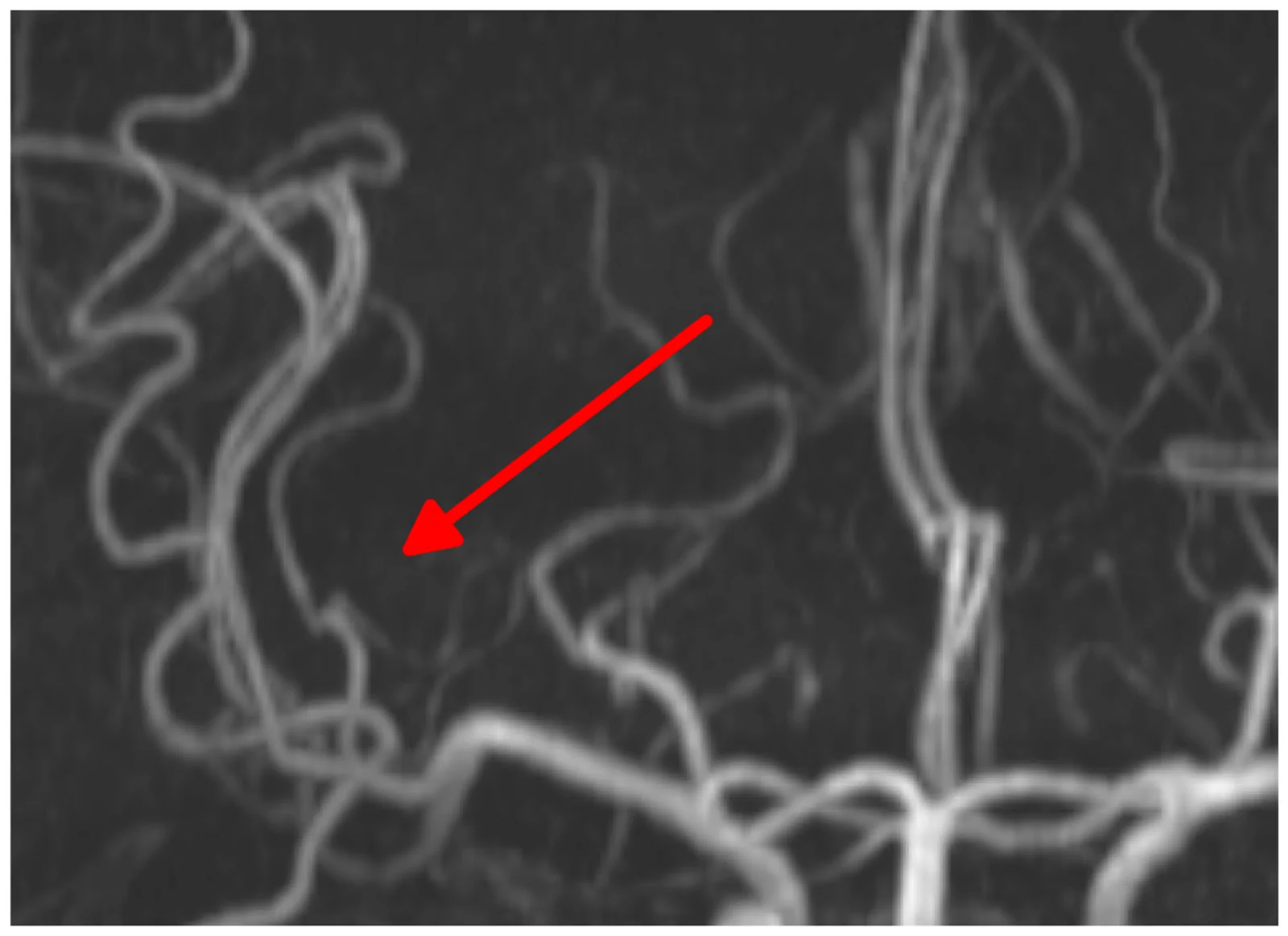
Representative false-positive candidate suppressed by Model-B. Maximum-intensity-projection (MIP) TOF-MRA image of a single scan acquired at 3.0 T. Model-A generated a false-positive candidate location in the region of the middle cerebral artery (MCA; arrow), whereas Model-B produced no candidate at this location. No aneurysm was present at this location according to the ground-truth annotation. The annotation marks the Model-A candidate only; Model-B produced no corresponding mark.

All false-positive findings were inspected in terms of their location. The locations of false-positive findings were compared between the models, Model-A and Model-B. Figure 2 shows the distribution of locations normalized by the total number of false positives from Model-A, expressed as a percentage. The figure visually compares the reduction of false positives and their locational trends between Model-A and Model-B. Figure 2 also shows the distribution of true-positive findings for comparison. The ICA region had the highest number of false positives (47.1% and 34.5% for Model-A and Model-B, respectively), with a clear reduction in Model-B. The MCA region (the second most frequent site of aneurysms, as shown in Table 1) had a lower false-positive percentage (11.3% and 7.7% for Model-A and Model-B, respectively). A representative example is shown in Figure 3, in which a false-positive candidate generated by Model-A in the MCA region was not produced by Model-B. For the ICPC and ACOM locations, the trends in false positives and true positives were similar, with a slight reduction observed for Model-B. BA, VA, and ACA locations all had lower percentages of false positives compared to the percentages of true positives. Details of the regional distributions of FP findings are presented in Table 4.

Regional analysis of FPs further demonstrated location-specific differences between the two models. Two-sided Wilcoxon signed-rank tests were performed for each vascular location to assess whether Model-B yielded fewer FPs than Model-A. After Holm adjustment for the eight regional comparisons, the reductions remained statistically significant in the ICA (adjusted *p* < 0.001) and MCA (adjusted *p* = 0.003), whereas the ACOM (adjusted *p* = 0.088), ICPC (adjusted *p* = 0.214), BA and VA (adjusted *p* = 0.500 each), and ACA and Other (adjusted *p* = 1.000 each) did not. The numbers of false positives in the BA and VA territories were small (13 → 9 and 6 → 2, respectively), with only four scans showing a non-zero difference at each location. With four such scans, the exact test can yield only three distinct two-sided *p* values (0.125, 0.625, and 1.0), the smallest of which already exceeds 0.05 before adjustment for multiplicity, so significance is unattainable at these locations regardless of the observed data. The corresponding results are presented descriptively. These findings indicate that the reduction in FP burden with Model-B varied according to vascular location. For the ICA and MCA, the mean numbers of false positives per scan were 0.547 and 0.131 for Model-A and 0.401 and 0.089 for Model-B, with mean paired differences of 0.146 (bootstrap 95% CI, 0.099 to 0.193) and 0.042 (0.020 to 0.067). Per-scan counts were zero in most scans, so the medians were zero at both locations.

**Figure 4:**
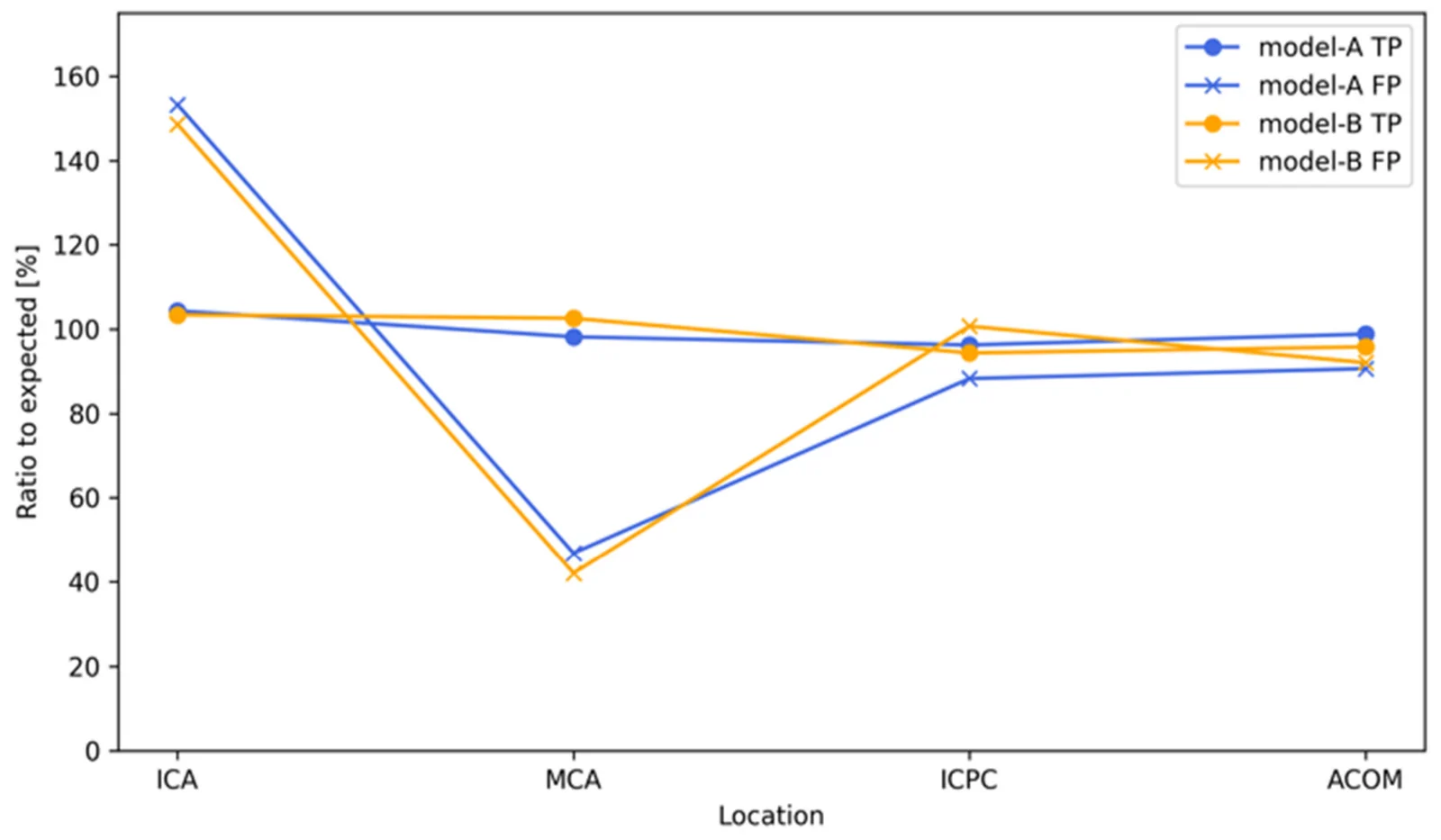
Distribution of TP and FP ratios normalized by expected counts from the ground-truth distribution. Observed-to-expected ratios of findings across vascular locations for Model-A (blue) and Model-B (orange). True-positive (TP) ratios are shown with circles and false-positive (FP) ratios with crosses. The ratios represent observed counts relative to expected counts estimated from the distribution of ground-truth aneurysms at each location (expressed as percentages). The observed FP distributions were compared with the expected distributions derived from the ground-truth proportions using global chi-square goodness-of-fit tests, followed by Holm-adjusted post hoc exact binomial tests where the global test was significant; location-dependent sensitivity was assessed with an exact (Fisher–Freeman–Halton) test of the location × detection-status table.

Figure 4 shows the observed-to-expected ratios of true-positive (TP) and false-positive (FP) findings across vascular locations for both models. TP ratios were close to 100% across all locations, indicating that TP findings followed the distribution of ground-truth aneurysms. The exact test of the location × detection-status table was not significant for either model (Model-A, *p* = 0.206; Model-B, *p* = 0.172; Figure 4, Supplementary Table S4). In contrast, the FP distribution deviated significantly from expectation in both models (chi-square = 74.8 for Model-A and 54.8 for Model-B, df = 3, both *p* < 0.001). In the post hoc tests, FPs were overrepresented in the ICA (Model-A, 153.2%; Model-B, 148.6%; both Holm-adjusted *p* < 0.001) and underrepresented in the MCA (Model-A, 46.8%; Model-B, 42.0%; both Holm-adjusted *p* < 0.001), whereas the ICPC and ACOM did not differ significantly from expectation in either model (Supplementary Table S4).

Within the 78-scan subset used for anatomical characterization, the FPs identified by Model-A and Model-B (*n* = 147 and *n* = 103, respectively) were classified into four categories of anatomical status: normal anatomy, aneurysm or suspected aneurysm, other pathological conditions, and indeterminate. The distribution of the FP status across these categories was 110, 35, 1, and 1 for Model-A, and 73, 28, 1, and 1 for Model-B, as presented in Table 4.

All FPs classified into the three categories other than normal anatomy (aneurysm or suspected aneurysm, other pathological conditions, and indeterminate) were annotated as acceptable, whereas approximately half of the FPs classified as normal anatomy were considered acceptable (Model-A: 50.0%, Model-B: 52.1%) (Table 5).

## 4. Discussion

In this study, we analyzed a multicenter validation dataset of 404 TOF-MRA scans obtained from the Brain Dock system to compare two versions of an AI algorithm developed to identify candidate locations of UCAs. Specifically, we conducted (i) a comparative evaluation of the two models in terms of performance—including sensitivity, the number of FPs/case, and stratified analyses of FPs/case by anatomical location and magnetic field strength—and (ii) an analysis of qualitative changes in FPs associated with FP reduction. As a result, the algorithmic refinement significantly reduced false positives with similar point estimates of sensitivity, and further revealed qualitative changes in the anatomical distribution and patterns of false-positive findings.

In this multicenter retrospective study, we validated a novel AI system equipped with a cluster-based post-processing algorithm designed to reduce false positives in UCA screening. The key finding was that the updated model (Model-B) achieved a 24% reduction in false positives per case (0.879 vs. 1.16) in the multicenter validation set, with aneurysm-wise sensitivity of 93.9% compared to the baseline model’s 94.3%. This finding suggests that a clinically acceptable balance can be achieved in addressing the well-recognized trade-off between sensitivity and specificity in AI-based identification of candidate locations of UCAs. Furthermore, our anatomical analysis revealed that the algorithm effectively suppresses common mimics in the internal carotid and middle cerebral arteries, which are frequent sources of “false-positive candidates” in AI-based identification of candidate locations.

The reduction of FPs from 1.16 to 0.879 per case corresponds to fewer candidate marks presented to readers in the context of mass screening (e.g., the Brain Dock system in Japan). In a low-prevalence population, the positive predictive value (PPV) of diagnostic tests is inherently low [26,27,28]. High FP rates exacerbate this issue, leading to “alert fatigue” for radiologists and potentially triggering unnecessary secondary imaging (e.g., CTA/DSA) or patient anxiety [29,30]. By filtering out approximately one-fourth of false-positive candidate findings, the updated model reduces the algorithmic false-positive burden presented to readers. Whether this reduction translates into shorter reading times, fewer downstream examinations, reduced patient anxiety, or improved cost-effectiveness was not assessed in this study and requires prospective evaluation. In absolute terms, the reduction corresponds to 0.28 fewer false-positive findings per scan, or approximately 280 fewer candidate marks per 1000 examinations.

From a neurosurgical perspective, false-positive candidate findings differ qualitatively in their downstream consequences. A candidate finding in the ICA cavernous segment, for example, rarely prompts additional imaging or intervention regardless of its label, whereas a candidate at an MCA bifurcation typically drives a decision on whether to proceed to CTA, DSA, or clinical follow-up. The observed anatomical concentration of the false-positive reduction in the ICA and MCA (Table 3) is therefore not a homogeneous 24% reduction from a clinical-action standpoint: the reductions in territories where flagged findings more often trigger downstream investigation may be particularly relevant to the referral rate from Brain Dock to specialist evaluation. This anatomically differentiated impact warrants prospective investigation in linked screening-and-referral datasets.

Model-B demonstrates highly competitive performance relative to established meta-analytic benchmarks for AI-mediated aneurysm detection. For instance, a systematic review by Din et al. reported a pooled sensitivity of 91.2% (95% CI: 82.2–95.8%) and a false-positive rate of 16.5% [18], while underscoring excessive FPs as a major barrier to clinical implementation. These findings are consistent with subsequent meta-analyses by Delfan et al. (90% lesion-wise sensitivity) [31] and Zhou et al. (0.87 pooled sensitivity; 95% CI: 0.835–0.91) [32]. Model-B’s sensitivity of 93.9% was within the range reported in prior meta-analyses, and its FP burden was 0.88 per case. Direct comparison should be interpreted cautiously because FP definitions differ across studies. In addition, both models cap the output at four candidates per scan, which bounds FPs per case by construction and limits direct comparison with benchmarks derived from systems without an output cap.

A critical finding of this study was that Model-B achieved a 24% reduction in FPs per case, with a scan-level negative predictive value of 94.1% in this enriched validation set. Applying the same prevalence-weighted approach, the projected negative predictive value at assumed prevalences of 2%, 3%, and 6% is 99.9%, 99.9%, and 99.7% for Model-A and 99.9%, 99.8%, and 99.6% for Model-B. Although a slight decrease in aneurysm-wise sensitivity was observed (from 94.3% to 93.9%), this difference was small and was mainly related to very small aneurysms (<3 mm). In large-scale screening settings such as the Japanese Brain Dock, radiologists may need to interpret a large number of cases; whether a reduction in the number of candidate marks affects the interpretive burden was not assessed in this study. However, the clinical implications of missed small aneurysms require careful consideration, and AI outputs should be interpreted as decision-support information rather than as a replacement for expert review [33,34]. The validation set was constructed with an approximately 1:1 ratio of aneurysm-positive to aneurysm-negative scans, whereas the prevalence of unruptured cerebral aneurysms in a screening population is only a few percent. As an illustrative extrapolation, we therefore projected the expected number of findings per 1000 examinations at assumed prevalences of 2%, 3%, and 6%, weighting the false-positive rates observed separately in aneurysm-negative and aneurysm-positive scans (Supplementary Table S5). At an assumed prevalence of 3%, the projection gives 1181 false-positive findings per 1000 examinations for Model-A and 955 for Model-B, with 29 of the 30 assumed aneurysm-positive individuals flagged. These projections extrapolate per-scan behavior observed in an enriched cohort and do not represent measured performance in a screening population. Five of the seven discordant aneurysms were smaller than 3 mm, and all seven were imaged at 1.5 T; detection at 3.0 T was fully concordant. Together with the concordant detection of 227 of 245 aneurysms, this pattern indicates that the refinement chiefly altered the selection of borderline candidates among small aneurysms. The potential cost of false-positive reduction is therefore concentrated in aneurysms smaller than 3 mm, for which sensitivity is lowest for both models, and this trade-off warrants explicit consideration when deploying algorithmic changes in screening practice.

Our detailed inspection of the dataset clarified how the model achieved this improvement. The significant reduction in ICA (26.7%) and MCA (32.1%) false positives suggests that the secondary classifier successfully learned to discriminate complex vascular features from true aneurysms based on local cluster morphology. The ICA is particularly prone to FPs owing to its tortuous course, the presence of infundibular dilatations at branching points, and overlapping venous structures—all of which can create focal signal-intensity clusters mimicking aneurysmal morphology on TOF-MRA. Similarly, MCA bifurcations and trifurcations generate geometrically complex configurations that challenge even expert readers, and the updated secondary classifier may have captured these anatomical characteristics to suppress non-aneurysmal candidates more selectively. After adjustment for multiple comparisons, the reduction was statistically supported in the ICA and MCA only. Such analyses that visualize where false positives are reduced are crucial for understanding AI behavior that cannot be fully captured by conventional performance metrics alone, and they provide a foundation for clinicians to appropriately interpret and apply AI outputs in real-world clinical practice. In an exploratory analysis, more than half of the remaining FPs were judged acceptable by the reviewing neurosurgeons (expert-perceived clinical acceptability). Because this outcome is inherently subjective and was assessed by single readers on non-overlapping subsets, it should be regarded as hypothesis-generating.

The concentration of discordant detections in aneurysms smaller than 3 mm is also clinically informative. Current Japanese Brain Dock guidelines and international recommendations generally do not indicate treatment for asymptomatic aneurysms < 3 mm in the absence of specific risk features, and management typically consists of follow-up imaging [7,34]. Consequently, a marginal loss in sensitivity concentrated in this size range has a different clinical implication from a comparable loss in medium or large aneurysms: the primary consequence is a delayed rather than missed opportunity for surveillance. This does not eliminate the concern—natural history data support ongoing follow-up of small aneurysms—but it does place the observed trade-off in a clinically calibrated context that should be considered when deploying algorithmic changes in screening practice.

These findings often corresponded to normal anatomical variants or dilatations rated as warranting confirmation.

Recent studies have shown that the sensitivity of AI systems for identifying candidate locations of UCAs is approaching the level of expert human readers [13,18,35,36]. In contrast, it has become increasingly evident that the major barrier to clinical implementation is a high false-positive burden [19,20,37,38]. Both the present study and our previous work support a paradigm shift in UCA research—from an exclusive focus on maximizing sensitivity toward optimizing overall clinical usability in identifying candidate locations [9]. Future progress will require more than incremental improvements in network architecture or increases in training data volume; greater emphasis should be placed on multi-stage decision frameworks, post-processing algorithms, integration of anatomical knowledge, and evaluation metrics that account for compatibility with real-world clinical workflows. In addition, evaluation frameworks incorporating qualitative analyses of false positives, as demonstrated in this study, can help visualize the model refinement process and provide guidance for comparing and selecting AI systems in an era where multiple AI models coexist. Overall, this study demonstrates—through multicenter validation—that a clinically oriented refinement strategy centered on false-positive reduction is effective for AI-based identification of candidate locations of unruptured intracranial aneurysms. Future work should focus on prospective studies and validation in real-world clinical settings to better assess how AI can complement clinicians’ decision-making.

### Limitations

Our study has several limitations. First, because this study was retrospective in design, prospective validation in a real-world clinical workflow is necessary to assess the actual impact on reading time and diagnostic accuracy. Second, the ground truth was established by expert review; however, small aneurysms (<3 mm) remain challenging to annotate consistently, even for experts. Third, we used a distance threshold corresponding to the aneurysm radius plus a 5 mm margin to define true positives. Although this criterion may be considered lenient for very small aneurysms (<3 mm), it is consistent with prior AI-based studies on candidate aneurysm localization and was applied identically to both models; therefore, the relative comparison between models remains valid. Fourth, the assessment of expert-perceived clinical acceptability for FPs was conducted by two neurosurgeons who reviewed distinct, non-overlapping subsets. Consequently, formal inter-rater reliability metrics, such as Cohen’s kappa, could not be quantified, potentially introducing inter-observer variability. Although this remains a common constraint in large-scale retrospective multicenter studies, future prospective research should employ dual-reader protocols with overlapping case assignments to facilitate robust agreement analysis. Fifth, per-institution performance is reported descriptively (Supplementary Table S7); the study was not powered for formal assessment of between-institution heterogeneity. Sixth, age, sex, and other demographic variables were not included in the anonymized dataset provided to the investigators, and acquisitions were concentrated in particular years that largely coincide with individual institutions; model fairness across sociodemographic groups and stability across acquisition eras could therefore not be assessed. Seventh, the validation cohort was enriched to an approximately 1:1 ratio of aneurysm-positive to aneurysm-negative scans; prevalence-dependent quantities such as predictive values do not transfer to a screening population with a prevalence of 2–6%, and the projections per 1000 examinations provided in the Discussion are illustrative rather than empirical. In addition, some findings scored as false positives were judged “aneurysm or suspected aneurysm” in the exploratory characterization (Table 4), indicating that the reference standard—restricted by design to saccular aneurysms ≥ 2 mm—may not capture all aneurysm-like lesions. This affects the absolute false-positive rates and sensitivities and, if the two models differ in their propensity to flag such lesions, could also affect the between-model comparison. Resolving this would require information beyond that available for the present dataset, and this residual uncertainty remains. The fixed four-candidate output cap right-truncates per-scan false-positive counts, which constrains the distributions underlying the paired tests and cross-study comparisons.

## 5. Conclusions

In this multicenter retrospective evaluation, a secondary cluster-filtering algorithm reduced the number of AI-generated false-positive candidate findings per scan by 24% (from 1.16 to 0.88), with similar point estimates of aneurysm-wise sensitivity (94.3% vs. 93.9%; paired difference −0.4 percentage points, 95% CI −3.1 to +2.1). These results demonstrate a reduction in the algorithmic false-positive burden of AI-based candidate identification. Whether this reduction improves reading workflow, downstream resource use, or patient outcomes was not assessed and requires prospective evaluation in true screening populations.

Future evaluations linking algorithmic false-positive reduction to downstream referral rates, reading time, and radiologist agreement will be needed to translate the observed algorithmic gains into measurable clinical benefit within existing Brain Dock workflows.

## Figures and Tables

**Table 1:** Characteristics of aneurysms in the dataset.

	Number of Aneurysms	True-Positive Locations and Sensitivity	
		Model-A	Model-B
Total aneurysms	245	231 (94.3)	230 (93.9)
Size of aneurysm (mm)			
<3	86 (35.1)	76 (88.4)	75 (87.2)
3.0 ≤ size < 5.0	60 (24.5)	58 (96.7)	57 (95.0)
5.0 ≤ size < 10	92 (37.6)	90 (97.8)	91 (98.9)
≥10	7 (2.9)	7 (100)	7 (100)
Location of aneurysm			
ICA	70 (28.6)	69 (98.6)	68 (97.1)
MCA	55 (22.4)	51 (92.7)	53 (96.4)
ICPC	44 (18.0)	40 (90.9)	39 (88.6)
ACOM	30 (12.2)	28 (93.3)	27 (90.0)
BA	22 (9.0)	22 (100)	22 (100)
VA	16 (6.5)	14 (87.5)	14 (87.5)
ACA	7 (2.9)	7 (100)	7 (100)
Other	1 (0.4)	0 (0)	0 (0)
Magnetic field strength			
1.5 T	188 (76.7)	177 (94.1)	176 (93.6)
3.0 T	57 (23.3)	54 (94.7)	54 (94.7)

ACA, anterior cerebral artery; ACOM, anterior communicating artery; BA, basilar artery; ICA, internal carotid artery; ICPC, internal carotid–posterior communicating artery; MCA, middle cerebral artery; VA, vertebral artery.

**Table 2:** Performance comparison of Model-A and Model-B.

	Model-A	Model-B	*p* Value
Sensitivity (%)	94.3	93.9	1.00
95% CI	[90.6, 96.8]	[90.1, 96.5]	
FPs per case	1.16	0.879	<0.001
Negative predictive value (%)	95.6	94.1	—

CI, confidence interval; FP, false positive; NPV, negative predictive value. Sensitivity was compared using McNemar’s test, FPs per case using the Wilcoxon signed-rank test. The negative predictive value is prevalence-dependent and applies to the composition of this validation set.

**Table 3:** Aneurysms detected by only one of the two models.

Detected By	Location	Size (mm)	Field Strength
Model-A only	ICA	2.3	1.5 T
	ICPC	2.6	1.5 T
	ACOM	2.8	1.5 T
	VA	4.6	1.5 T
Model-B only	MCA	2.3	1.5 T
	VA	2.5	1.5 T
	MCA	5.6	1.5 T

ACOM, anterior communicating artery; ICA, internal carotid artery; ICPC, internal carotid–posterior communicating artery; MCA, middle cerebral artery; T, tesla; VA, vertebral artery.

**Table 4:** Regional distribution of false-positive findings and statistical comparison between Model-A and Model-B.

Location	Aneurysms, *n* (%)	Model-A			Model-B			Reduction		*p* Value	*p* Value (Holm)
		FPs (*n*)	% of Model-A	FPs/case	FPs (*n*)	% of Model-A	FPs/case	Absolute (*n*)	Relative (%)		
ICA	70 (28.6%)	221	47.1%	0.547	162	34.5%	0.401	59	26.7%	<0.001	<0.001 *
MCA	55 (22.4%)	53	11.3%	0.131	36	7.7%	0.089	17	32.1%	<0.001	0.003 *
ICPC	44 (18.0%)	80	17.1%	0.198	69	14.7%	0.171	11	13.8%	0.043	0.214
ACOM	30 (12.2%)	56	11.9%	0.139	43	9.2%	0.106	13	23.2%	0.015	0.088
BA	22 (9.0%)	13	2.8%	0.032	9	1.9%	0.022	4	30.8%	0.125	0.500
VA	16 (6.5%)	6	1.3%	0.015	2	0.4%	0.005	4	66.7%	0.125	0.500
ACA	7 (2.9%)	16	3.4%	0.040	14	3.0%	0.035	2	12.5%	0.754	1.000
Other	1 (0.4%)	24	5.1%	0.059	20	4.3%	0.050	4	16.7%	0.559	1.000
Total	245	469	100.0%	1.161	355	75.7%	0.879	114	24.3%	<0.001	—

ACA, anterior cerebral artery; ACOM, anterior communicating artery; BA, basilar artery; FP, false positive; ICA, internal carotid artery; ICPC, internal carotid–posterior communicating artery; MCA, middle cerebral artery; VA, vertebral artery; —, not applicable. * Statistically significant after Holm adjustment (adjusted *p* < 0.05). *p* values were calculated using the two-sided Wilcoxon signed-rank test, with exact *p* values obtained from the conditional sign-flip distribution. The regional analyses are exploratory; adjusted *p* values were obtained with the Holm procedure over the eight regional comparisons, which do not include the comparison for the whole cohort. The number of scans with a non-zero difference between the models was 79 (ICA), 23 (MCA), 22 (ICPC), 25 (ACOM), 4 (BA), 4 (VA), 10 (ACA), and 23 (Other). % of Model-A, percentage of the total number of false-positive findings generated by Model-A.

**Table 5:** Anatomical classification and expert-perceived clinical acceptability of false-positive findings for Model-A and Model-B (exploratory analysis).

FP Categories	Model-A			Model-B		
	FPs (*n*)	FPs (%)	Acceptable (%)	FPs (*n*)	FPs (%)	Acceptable (%)
Normal anatomy	110	74.8	50.0	73	70.9	52.1
Aneurysm or suspected aneurysm	35	23.8	100	28	27.2	100
Other pathological conditions	1	0.7	100	1	1.0	100
Indeterminate	1	0.7	100	1	1.0	100
Total	147	100	62.6	103	100	66.0

FP, false positive. Acceptable FPs comprised all cases classified as aneurysm or suspected aneurysm, other pathological conditions, or indeterminate, as well as approximately half of those classified as normal anatomy. Findings classified as “aneurysm or suspected aneurysm” include lesions outside the evaluation targets defined in Section 2.2 and do not necessarily indicate an omission from the ground truth.

## Data Availability

The data analyzed in this study are not publicly available and cannot be shared. The statistical analysis code used in this study is available from the corresponding author upon reasonable request. The evaluated AI models are proprietary commercial software; their source code, model weights, and training data are not available.

## Supplementary Materials

The following supporting information can be downloaded at: https://www.mdpi.com/article/10.3390/jcm15187259/s1, Supplementary Table S1: MRI scanner characteristics and acquisition parameters obtained from the DICOM metadata; Supplementary Table S2: False-positive findings by magnetic field strength for Model-A and Model-B; Supplementary Table S3: Detection sensitivity by magnetic field strength and aneurysm size for Model-A and Model-B; Supplementary Table S4: Observed and expected true-positive and false-positive findings for the four principal aneurysm locations and each model. TP and FP counts are shown alongside the expected values based on the distribution of ground-truth aneurysms, the ratios of observed to expected counts (in %), and the corresponding *p* values. For FP findings, the observed distribution across the four locations was compared with the expected distribution using a chi-square goodness-of-fit test, followed by Holm-adjusted post-hoc exact binomial tests for each location; TP findings were assessed using an exact test of the association between location and detection status. Model-A and Model-B results are presented separately to allow comparison of performance across locations. The exact test of the location × detection-status table was not significant for either model (Model-A, *p* = 0.206; Model-B, *p* = 0.172; exact Fisher–Freeman–Halton test), so TP *p* values are not shown per location; Supplementary Table S5: Projected findings per 1000 examinations at assumed screening prevalences. Counts were projected by weighting the rates observed separately in aneurysm-negative and aneurysm-positive scans by the assumed prevalence; the number of individuals with at least one detected aneurysm was derived from the scan-wise sensitivity of 96.1%; Supplementary Table S6: Effect of the spatial matching margin on detection sensitivity and false-positive burden. All model outputs were re-scored with the margin added to the aneurysm radius set to 5, 4, and 3 mm; the 5-mm margin is the criterion used in the main analysis, and the 4-mm and 3-mm margins are stricter conditions applied for comparison; Supplementary Table S7: Performance of Model-A and Model-B by participating institution. Results are presented descriptively; the study was not powered for formal between-institution comparison; Supplementary Material S2: Completed TRIPOD + AI checklist [25].

## Abbreviations

The following abbreviations are used in this manuscript:

UCAs	unruptured cerebral aneurysms
MRI	magnetic resonance imaging
MRA	magnetic resonance angiography
TOF	time-of-flight
AI	artificial intelligence
FPs	false positives
TP	true positive
TN	true negative
FN	false negative
NPV	negative predictive value
CI	confidence interval
ICA	internal carotid artery
MCA	middle cerebral artery
ICPC	internal carotid–posterior communicating artery
ACOM	anterior communicating artery
ACA	anterior cerebral artery
BA	basilar artery
VA	vertebral artery
CNN	convolutional neural network
DICOM	Digital Imaging and Communications in Medicine
PMDA	Pharmaceuticals and Medical Devices Agency
MIP	maximum-intensity projection
CTA	computed tomography angiography
DSA	digital subtraction angiography
PPV	positive predictive value
3D	three-dimensional
