# Supplementary material for: Clinical Comparison of Two Versions of a Commercial Artificial Intelligence System for Identifying Candidate Locations of Cerebral Aneurysms: Reduction and Characterization of False-Positive Findings
Source: J Clin Med. 2026 Sep 18;15(18):7259. doi: 10.3390/jcm15187259 (PMC13607247; doi:10.3390/jcm15187259)
Supplement: Supplementary file 1 [file jcm-15-07259-s001.zip › Supplementary Material S2.pdf]

TRIPOD + AI Checklist

Clinical Comparison of Two Versions of a Commercial Artificial Intelligence System for Identifying Candidate Locations of Cerebral Aneurysms: Reduction and Characterization of False-Positive Findings

Journal of Clinical Medicine (submitted). Page numbers refer to the submitted manuscript.

This study is an external evaluation (validation) of two existing, commercially available models. Items marked "D" (development only) are therefore not applicable.

Section/Topic	Item	Development / evaluation	Checklist item	Reported on page
**TITLE**				
Title	1	D;E	Identify the study as developing or evaluating the performance of a multivariable prediction model, the target population, and the outcome to be predicted	1
**ABSTRACT**				
Abstract	2	D;E	See TRIPOD+AI for Abstracts checklist	1
**INTRODUCTION**				
Background	3a	D;E	Explain the healthcare context (including whether diagnostic or prognostic) and rationale for developing or evaluating the prediction model, including references to existing models	2
	3b	D;E	Describe the target population and the intended purpose of the prediction model in the context of the care pathway, including its intended users (e.g., healthcare professionals, patients, public)	2
	3c	D;E	Describe any known health inequalities between sociodemographic groups	2 (ethnic difference in rupture risk: approximately 2.8-fold higher in Japanese than in Western populations). No other sociodemographic inequalities were assessed
Objectives	4	D;E	Specify the study objectives, including whether the study describes the development or validation of a prediction model (or both)	2 (evaluation/validation only)
**METHODS**				
Data	5a	D;E	Describe the sources of data separately for the development and evaluation datasets (e.g., randomised trial, cohort, routine care or registry data), the rationale for using these data, and representativeness of the data	3
	5b	D;E	Specify the dates of the collected participant data, including start and end of participant accrual; and, if applicable, end of follow-up	3 (Section 2.2; accrual period stated. Acquisitions were concentrated in particular years, and acquisition year largely coincides with the contributing institution, so an era-stratified comparison was not performed)
Participants	6a	D;E	Specify key elements of the study setting (e.g., primary care, secondary care, general population) including the number and location of centres	3 (Brain Dock screening; seven institutions in Japan)
	6b	D;E	Describe the eligibility criteria for study participants	3, 4 (Figure 1), 5
	6c	D;E	Give details of any treatments received, and how they were handled during model development or evaluation, if relevant	NA – asymptomatic screening population; previously treated subjects were outside the scope of data collection (see 6b)
Data preparation	7	D;E	Describe any data pre-processing and quality checking, including whether this was similar across relevant sociodemographic groups	3 (anonymization only; no quality-based exclusion), 5 (all processing internal to the software)
Outcome	8a	D;E	Clearly define the outcome that is being predicted and the time horizon, including how and when assessed, the rationale for choosing this outcome, and whether the method of outcome assessment is consistent across sociodemographic groups	4, 5 (only saccular aneurysms ≥ 2 mm were evaluation targets), 6
	8b	D;E	If outcome assessment requires subjective interpretation, describe the qualifications and demographic characteristics of the outcome assessors	4 (Section 2.2; three board-certified radiologists or neurosurgeons with at least 5 years of experience in diagnosing cerebral aneurysms, employed by medical or academic institutions; each scan reviewed by one or two of them, with disagreements resolved by consensus discussion)
	8c	D;E	Report any actions to blind assessment of the outcome to be predicted	4 (Section 2.2; annotators were blinded to the AI outputs and knew only whether a scan had been reported as aneurysm-positive or aneurysm-negative)
Predictors	9a	D	Describe the choice of initial predictors (e.g., literature, previous models, all available predictors) and any pre-selection of predictors before model building	NA – development item; this study is an evaluation (external validation) of two existing models
	9b	D;E	Clearly define all predictors, including how and when they were measured (and any actions to blind assessment of predictors for the outcome and other predictors)	3, 5 (the input is the TOF-MRA volume itself; scanner and acquisition parameters in Supplementary Table 1)
	9c	D;E	If predictor measurement requires subjective interpretation, describe the qualifications and demographic characteristics of the predictor assessors	NA (predictors are the imaging data itself)
Sample size	10	D;E	Explain how the study size was arrived at (separately for development and evaluation), and justify that the study size	3 (Section 2.2)

			was sufficient to answer the research question. Include details of any sample size calculation	
Missing data	11	D;E	Describe how missing data were handled. Provide reasons for omitting any data	3 (explicit statement that there were no missing imaging data or outcome annotations among the 404 included scans), 4 (Figure 1: 2 of 406 scans excluded, with reason)
Analytical methods	12a	D	Describe how the data were used (e.g., for development and evaluation of model performance) in the analysis, including whether the data were partitioned, considering any sample size requirements	NA – development item; this study is an evaluation (external validation) of two existing models
	12b	D	Depending on the type of model, describe how predictors were handled in the analyses (functional form, rescaling, transformation, or any standardisation)	NA – development item; this study is an evaluation (external validation) of two existing models
	12c	D	Specify the type of model, rationale, all model-building steps, including any hyperparameter tuning, and method for internal validation	NA – development item; this study is an evaluation (external validation) of two existing models
	12d	D;E	Describe if and how any heterogeneity in estimates of model parameter values and model performance was handled and quantified across clusters (e.g., hospitals, countries). See TRIPOD-Cluster for additional considerations	9, 11 (Sections 3.4, 2.6; performance quantified separately by magnetic field strength and, descriptively, by participating institution. The study was not powered for formal assessment of between-institution heterogeneity; this is stated in the Limitations)
	12e	D;E	Specify all measures and plots used (and their rationale) to evaluate model performance (e.g., discrimination, calibration, clinical utility) and, if relevant, to compare multiple models	6–8, 12–14 (Sections 2.6, 3.1–3.6; sensitivity, FPs per case, NPV, qualitative FP classification, observed-to-expected ratios, Tables 3–4, Figure 4; a sensitivity analysis of the spatial matching margin at 5, 4, and 3 mm; exact two-sided p values for the paired comparisons of FPs were computed from the conditional sign-flip distribution rather than the normal approximation; the eight region-wise comparisons were adjusted with the Holm procedure, and the distribution of false positives across the four principal locations was assessed with a chi-square goodness-of-fit test followed by Holm-adjusted post-hoc exact binomial tests; the region-wise analyses and the assessment of expert-perceived clinical acceptability are reported as exploratory)
	12f	E	Describe any model updating (e.g., recalibration) arising from the model evaluation, either overall or for particular sociodemographic groups or settings	NA – no model updating or recalibration was performed by the investigators; Model-B is a pre-existing updated version released by the manufacturer
	12g	E	For model evaluation, describe how the model predictions were calculated (e.g., formula, code, object, application programming interface)	5 (Sections 2.3, 2.4; three-step pipeline: vessel extraction and key-point detection, CNN-based confidence scoring of 3D patches, and cluster selection; Model-B adds a secondary cluster-classification model. The output is capped at four candidate locations per scan, which right-truncates per-scan false-positive counts)
Class imbalance	13	D;E	If class imbalance methods were used, state why and how this was done, and any subsequent methods to recalibrate the model or the model predictions	3 (the evaluation dataset was deliberately balanced to approximately 50% aneurysm-positive and 50% aneurysm-negative scans; no recalibration was applied)
Fairness	14	D;E	Describe any approaches that were used to address model fairness and their rationale	16 (Limitations; age, sex, and other demographic variables were not available, so fairness across sociodemographic groups could not be assessed)
Model output	15	D	Specify the output of the prediction model (e.g., probabilities, classification). Provide details and rationale for any classification and how the thresholds were identified	NA – development item; this study is an evaluation (external validation) of two existing models
Training versus evaluation	16	D;E	Identify any differences between the development and evaluation data in healthcare setting, eligibility criteria, outcome, and predictors	3 (none of the evaluation cases were used for training, and all were obtained from institutions different from those contributing the training data)
Ethical approval	17	D;E	Name the institutional research board or ethics committee that approved the study and describe the participant-informed consent or the ethics committee waiver of informed consent	3, 17
**OPEN SCIENCE**				
Funding	18a	D;E	Give the source of funding and the role of the funders for the present study	17 (Funding)
Conflicts of interest	18b	D;E	Declare any conflicts of interest and financial disclosures for all authors	18 (Conflicts of Interest)
Protocol	18c	D;E	Indicate where the study protocol can be accessed or state that a protocol was not prepared	17 (Institutional Review Board Statement)
Registration	18d	D;E	Provide registration information for the study, including register name and registration number, or state that the study was not registered	17 (the study was not registered in a clinical trial registry)
Data sharing	18e	D;E	Provide details of the availability of the study data	17 (Data Availability Statement)
Code sharing	18f	D;E	Provide details of the availability of the analytical code	17 (Data Availability Statement)
**PATIENT & PUBLIC INVOLVEMENT**				

Patient & Public Involvement	19	D;E	Provide details of any patient and public involvement during the design, conduct, reporting, interpretation, or dissemination of the study or state no involvement.	17
**RESULTS**				
Participants	20a	D;E	Describe the flow of participants through the study, including the number of participants with and without the outcome and, if applicable, a summary of the follow-up time. A diagram may be helpful.	4 (Figure 1), 8 (404 scans: 207 aneurysm-positive with 245 aneurysms, 197 aneurysm-negative)
	20b	D;E	Report the characteristics overall and, where applicable, for each data source or setting, including the key dates, key predictors (including demographics), treatments received, sample size, number of outcome events, follow-up time, and amount of missing data. A table may be helpful. Report any differences across key demographic groups.	4–5 (Table 1), Supplementary Table 1, 16 (Section 2.2, Limitations; age and sex not available)
	20c	E	For model evaluation, show a comparison with the development data of the distribution of important predictors (demographics, predictors, and outcome).	17 (commercial product; training data not available for comparison)
Model development	21	D;E	Specify the number of participants and outcome events in each analysis (e.g., for model development, hyperparameter tuning, model evaluation)	4–5, 8–9 (Tables 1, 2), 12 (Table 3), 14 (Table 4)
Model specification	22	D	Provide details of the full prediction model (e.g., formula, code, object, application programming interface) to allow predictions in new individuals and to enable third-party evaluation and implementation, including any restrictions on access or re-use (e.g., freely available, proprietary)	NA – development item; this study is an evaluation (external validation) of two existing models. The evaluated software is proprietary (EIRL aneurysm, approval number 30100BZX00142000); the model is described at the algorithm level on p. 5
Model performance	23a	D;E	Report model performance estimates with confidence intervals, including for any key subgroups (e.g., sociodemographic). Consider plots as presentation.	4–5 (Table 1), 8–9 (Table 2), 9 (subgroup results), 12 (Table 3), 13 (Figure 4), Supplementary Tables 2–7 (Sections 3.1–3.6; including the paired difference in sensitivity with a score-based confidence interval and bootstrap confidence intervals for the paired differences in FPs per scan)
	23b	D;E	If examined, report results of any heterogeneity in model performance across clusters. See TRIPOD Cluster for additional details.	9, 11 (Section 3.4; by magnetic field strength, Supplementary Table 2; by participating institution, Supplementary Table 7)
Model updating	24	E	Report the results from any model updating, including the updated model and subsequent performance	NA – no model updating was performed by the investigators
**DISCUSSION**				
Interpretation	25	D;E	Give an overall interpretation of the main results, including issues of fairness in the context of the objectives and previous studies	14–16
Limitations	26	D;E	Discuss any limitations of the study (such as a non-representative sample, sample size, overfitting, missing data) and their effects on any biases, statistical uncertainty, and generalizability	16
Usability of the model in the context of current care	27a	D	Describe how poor quality or unavailable input data (e.g., predictor values) should be assessed and handled when implementing the prediction model	NA – development item; this study is an evaluation (external validation) of two existing models
	27b	D	Specify whether users will be required to interact in the handling of the input data or use of the model, and what level of expertise is required of users	NA – development item; this study is an evaluation (external validation) of two existing models. The intended use as decision support alongside expert review is stated on p. 15
	27c	D;E	Discuss any next steps for future research, with a specific view to applicability and generalizability of the model	16

From: Collins GS, Moons KGM, Dhiman P, et al. BMJ 2024;385:e078378. doi:10.1136/bmj-2023-078378
