# Supplementary material for: Clinical Comparison of Two Versions of a Commercial Artificial Intelligence System for Identifying Candidate Locations of Cerebral Aneurysms: Reduction and Characterization of False-Positive Findings
Source: J Clin Med. 2026 Sep 18;15(18):7259. doi: 10.3390/jcm15187259 (PMC13607247; doi:10.3390/jcm15187259)
Supplement: Supplementary file 1 [file jcm-15-07259-s001.zip › Supplementary Tables.pdf]

**Supplementary Table S1.** MRI scanner characteristics and acquisition parameters obtained from the DICOM metadata.

| Site | Vendor | Scanner | Field strength (T) | TE (ms) | Voxel size (mm) |
|---|---|---|---|---|---|
| 1 | GE MEDICAL SYSTEMS | Signa HDxt | 3.0 | 2.6 | 0.4 × 0.4 × 0.5 |
| 2 | GE MEDICAL SYSTEMS | Signa HDxt | 1.5 | 3.3 | 0.4 × 0.4 × 0.5 |
| 2 | GE MEDICAL SYSTEMS | GENESIS_SIGNA | 1.5 | 6.9 | 0.3 × 0.3 × 0.6 |
| 3 | SIEMENS | Avanto | 1.5 | 7.2 | 0.6 × 0.6 × 0.6 |
| 3 | SIEMENS | Avanto_fit | 1.5 | 7.2 | 0.2 × 0.2 × 0.5 |
| 4 | Philips | Prodiva CX | 1.5 | 6.9 | 0.5 × 0.5 × 0.6 |
| 5 | GE MEDICAL SYSTEMS | SIGNA Architect | 3.0 | 3.4 | 0.4 × 0.4 × 0.5 |
| 6 | SIEMENS | Avanto | 1.5 | 7.2 | 0.4 × 0.4 × 0.7 |
| 7 | TOSHIBA_MEC | MRT200SP5 | 1.5 | 6.8 | 0.4 × 0.4 × 0.5 |

TE, echo time; T, Tesla.

**Supplementary Table S2.** False-positive findings by magnetic field strength for Model-A and Model-B.

| Field strength | Scans, n | Model-A FPs, n | Model-A FPs/scan | Model-B FPs, n | Model-B FPs/scan | Relative reduction, % | p value |
|---|---|---|---|---|---|---|---|
| 1.5 T | 260 | 259 | 1.00 | 201 | 0.77 | 22.4 | < 0.001 |
| 3.0 T | 144 | 210 | 1.46 | 154 | 1.07 | 26.7 | < 0.001 |

FP, false positive. p values were calculated using the two-sided Wilcoxon signed-rank test, with exact p values obtained from the conditional sign-flip distribution.

**Supplementary Table S3.** Detection sensitivity by magnetic field strength and aneurysm size for Model-A and Model-B.

| Field strength | Aneurysm size (mm) | No. of aneurysms | Model-A, TP (sensitivity, %) | Model-B, TP (sensitivity, %) |
|---|---|---|---|---|
| 1.5 T | < 3 | 49 | 42 (85.7) | 41 (83.7) |
| | 3.0 ≤ size < 5.0 | 44 | 42 (95.5) | 41 (93.2) |
| | 5.0 ≤ size < 10 | 88 | 86 (97.7) | 87 (98.9) |
| | ≥ 10 | 7 | 7 (100) | 7 (100) |
| | Subtotal | 188 | 177 (94.1) | 176 (93.6) |
| 3.0 T | < 3 | 37 | 34 (91.9) | 34 (91.9) |
| | 3.0 ≤ size < 5.0 | 16 | 16 (100) | 16 (100) |
| | 5.0 ≤ size < 10 | 4 | 4 (100) | 4 (100) |
| | ≥ 10 | 0 | – | – |
| | Subtotal | 57 | 54 (94.7) | 54 (94.7) |

TP, true positive. Sensitivity (%) was calculated as the number of true positives divided by the number of aneurysms. Subtotals correspond to the totals for each field strength in Table 1. –, not applicable; no aneurysm ≥10 mm was imaged at 3.0 T.

**Supplementary Table S4.** Observed and expected true-positive and false-positive findings for the four principal aneurysm locations and each model. TP and FP counts are shown alongside the expected values based on the distribution of ground-truth aneurysms, the ratios of observed to expected counts (in %), and the corresponding p values. For FP findings, the observed distribution across the four locations was compared with the expected distribution using a chi-square goodness-of-fit test, followed by Holm-adjusted post-hoc exact binomial tests for each location; TP findings were assessed using an exact test of the association between location and detection status. Model-A and Model-B results are presented separately to allow comparison of performance across locations. The exact test of the location × detection-status table was not significant for either model (Model-A, p = 0.206; Model-B, p = 0.172; exact Fisher–Freeman–Halton test), so TP p values are not shown per location.

| Model | Location | GT | TP | Expected TP | TP ratio to expected (%) | FP | Expected FP | FP ratio to expected (%) | FP p value (Holm) |
|---|---|---|---|---|---|---|---|---|---|
| Model-A | ICA | 70 | 69 | 66.1 | 104.3 | 221 | 144.2 | 153.2 | < 0.001 |
| | MCA | 55 | 51 | 52.0 | 98.2 | 53 | 113.3 | 46.8 | < 0.001 |
| | ICPC | 44 | 40 | 41.6 | 96.2 | 80 | 90.7 | 88.2 | 0.424 |
| | ACOM | 30 | 28 | 28.3 | 98.8 | 56 | 61.8 | 90.6 | 0.448 |
| Model-B | ICA | 70 | 68 | 65.8 | 103.4 | 162 | 109.0 | 148.6 | < 0.001 |
| | MCA | 55 | 53 | 51.7 | 102.5 | 36 | 85.7 | 42.0 | < 0.001 |
| | ICPC | 44 | 39 | 41.3 | 94.3 | 69 | 68.5 | 100.7 | 1.000 |
| | ACOM | 30 | 27 | 28.2 | 95.8 | 43 | 46.7 | 92.0 | 1.000 |

ACOM, anterior communicating artery; FP, false positive; ICA, internal carotid artery; ICPC, internal carotid-posterior communicating artery; MCA, middle cerebral artery; TP, true positive. p values were calculated as described in the table caption.

**Supplementary Table S5.** Projected findings per 1,000 examinations at assumed screening prevalences. Counts were projected by weighting the rates observed separately in aneurysm-negative and aneurysm-positive scans by the assumed prevalence; the number of individuals with at least one detected aneurysm was derived from the scan-wise sensitivity of 96.1%.

| Prevalence (%) | Aneurysm-positive scans, n | Model-A FPs, n | Model-B FPs, n | Absolute reduction, n | Individuals with ≥1 detected aneurysm, n | Model-A NPV (%) | Model-B NPV (%) |
|---|---|---|---|---|---|---|---|
| 2 | 20 | 1182 | 956 | 226 | 19 | 99.9 | 99.9 |
| 3 | 30 | 1181 | 955 | 227 | 29 | 99.9 | 99.8 |
| 6 | 60 | 1180 | 950 | 230 | 58 | 99.7 | 99.6 |

FP, false positive; NPV, negative predictive value. The absolute reduction was obtained from the unrounded values, so at a prevalence of 3% it differs by 1 from the difference between the two rounded counts. These projections are illustrative extrapolations from an enriched cohort.

**Supplementary Table S6.** Effect of the spatial matching margin on detection sensitivity and false-positive burden. All model outputs were re-scored with the margin added to the aneurysm radius set to 5, 4, and 3 mm; the 5-mm margin is the criterion used in the main analysis, and the 4-mm and 3-mm margins are stricter conditions applied for comparison.

| Model | Aneurysm size (mm) | No. of aneurysms | Sensitivity at radius + 5 mm (%) | Sensitivity at radius + 4 mm (%) | Sensitivity at radius + 3 mm (%) | FPs/case at radius + 5 / 4 / 3 mm |
|---|---|---|---|---|---|---|
| Model-A | < 3 | 86 | 76 (88.4) | 74 (86.0) | 73 (84.9) | |
| | 3.0 ≤ size < 5.0 | 60 | 58 (96.7) | 58 (96.7) | 57 (95.0) | |
| | 5.0 ≤ size < 10 | 92 | 90 (97.8) | 90 (97.8) | 88 (95.7) | |
| | ≥ 10 | 7 | 7 (100) | 7 (100) | 7 (100) | |
| | All | 245 | 231 (94.3) | 229 (93.5) | 225 (91.8) | 1.161 / 1.168 / 1.178 |
| Model-B | < 3 | 86 | 75 (87.2) | 75 (87.2) | 73 (84.9) | |
| | 3.0 ≤ size < 5.0 | 60 | 57 (95.0) | 57 (95.0) | 55 (91.7) | |
| | 5.0 ≤ size < 10 | 92 | 91 (98.9) | 90 (97.8) | 89 (96.7) | |

| | | | | | |
|---|---|---|---|---|---|
| ≥ 10 | 7 | 7 (100) | 6 (85.7) | 5 (71.4) | |
| All | 245 | 230 (93.9) | 228 (93.1) | 222 (90.6) | 0.879 / 0.884 / 0.899 |

FP, false positive. Sensitivity values are shown as the number of detected aneurysms with the corresponding percentage in parentheses. The same criterion was applied identically to both models.

**Supplementary Table S7.** Performance of Model-A and Model-B by participating institution. Results are presented descriptively; the study was not powered for formal between-institution comparison.

| Institution | Scans, n | Aneurysms, n | Model-A sensitivity, n (%) | Model-B sensitivity, n (%) | Model-A FPs/case | Model-B FPs/case |
|---|---|---|---|---|---|---|
| 1 | 48 | 58 | 55 (94.8) | 55 (94.8) | 1.250 | 0.813 |
| 2 | 55 | 63 | 61 (96.8) | 62 (98.4) | 1.455 | 1.091 |
| 3 | 67 | 83 | 74 (89.2) | 72 (86.7) | 0.701 | 0.448 |
| 4 | 50 | 0 | n/a | n/a | 0.360 | 0.320 |
| 5 | 97 | 0 | n/a | n/a | 1.557 | 1.196 |
| 6 | 21 | 22 | 22 (100.0) | 22 (100.0) | 1.048 | 0.762 |
| 7 | 66 | 19 | 19 (100.0) | 19 (100.0) | 1.379 | 1.182 |
| Total | 404 | 245 | 231 (94.3) | 230 (93.9) | 1.161 | 0.879 |

FP, false positive. Sensitivity is aneurysm-wise and is shown as the number of detected aneurysms with the corresponding percentage in parentheses. Institutions 4 and 5 contributed only aneurysm-negative scans, so sensitivity is not defined for them. FPs per case are not directly comparable across institutions because the proportion of aneurysm-positive scans differs between institutions and the false-positive rate differs between aneurysm-negative and aneurysm-positive scans.
